# A Practical Method for Determination of Nine Nucleosides in *Tricholoma matsutake* by UPLC/MS and Quantitative Analysis of Multicomponents Using Single Marker Method

**DOI:** 10.1155/2021/9571329

**Published:** 2021-09-11

**Authors:** Li Yong, An-Qin Leng, Zhi-Xiang Yang, Ying Xue

**Affiliations:** Sichuan Provincial Center for Disease Control and Prevention, Chengdu 610041, China

## Abstract

Nucleosides can be used as quality evaluation indicators of *Tricholoma matsutake*. In this work, a new ultra-performance liquid chromatography-tandem mass spectrometry (UPLC/MS) strategy for quantitative analysis of multiple components using a single marker (QAMS) was proposed to determine nine nucleosides (adenosine, cytidine, guanosine, inosine, uridine, 2′-deoxyadenosine, 2′-deoxycytidine, 2′-deoxyguanosine, and 2′-deoxyuridine) in *T. matsutake*. Guanosine was set as the internal reference substance, whose content in *T. matsutake* was determined using the conventional external standard method. Relative correction factors (RCFs) between guanosine and eight other nucleosides were measured. The concentrations of the eight components were calculated with the obtained RCFs by QAMS. An ultrasonic extraction method is used for sample preparation. This method was validated to be sensitive, precise, and accurate with the LODs of 0.31–1.9 ng, the overall intraday and interday variations less than 4.08%, and the overall recovery over 89.0%. The correlation coefficients (*r*^2^) of the calibration curves were higher than 0.9918. The values of vector angle analysis were above 0.99845, which indicates no significant differences between the conventional external standard method and the present QAMS method. As far as we know, this is also the first report of UPLC/MS analysis of nucleoside compounds by QAMS, providing an efficient and feasible quality assessment method for other natural products containing nucleosides.

## 1. Introduction

*Tricholoma matsutake* is a wild edible fungus endemic to East Asia. The Hengduan Mountain Region of Southwest China, especially Sichuan Province and Yunnan Province, is the world's foremost production center of *T. matsutake*. *T. matsutake* is widely used not only in high-end foods for its unique flavor and fresh taste but also in health care products due to its antioxidant [1, 2], immunomodulatory [3, 4], and anti-tumor properties [5, 6].

Nucleic acid constituents that can regulate various physiological processes *in vivo* through the purine/pyrimidine receptors are considered suitable markers for the quality evaluation of *T. matsutake* [7, 8]. Sichuan Food and Drug Administration commissioned researchers from several units, including our group, to jointly draft local standards for the safety of *T. matsutake* and its products, which were implemented on July 20, 2018. On this basis, our team conducted an in-depth study on the active ingredients in *T. matsutake* and proposed for the first time that nucleic acid compounds be used as the quality control markers for commercial *T. matsutake* products. Subsequently, a validated UPLC/MS method based on the external standard method was developed to determine the content of each nucleic acid compound in 80 samples from different regions of Sichuan Province [7]. However, the external standard method, as a classical quantitative test method, requires the purchase of all reference substances and preparation of each corresponding solution as well as other operations, which may be considered wasteful of time and money. Therefore, it is desirable to develop a new, accurate, and rapid analytical method for the determination of nucleosides in *T. matsutake*.

Natural products often consist of a variety of chemical components with the same skeletal structure [9, 10]. The quantitative analysis of multiple components using a single marker (QAMS) method could reduce the complexity and cost of conventional external standard methods. QAMS is a new method for multicomponent quality evaluation, which has been successfully applied to food analysis to solve the problem of a shortage of reference materials and high cost in multicomponent analysis. It makes the multicomponent quality evaluation of natural products easier as well as more cost-effective [11]. At present, the QAMS method has been widely used in the quality control research of foods, including Rhizoma Paridis [11], oolong tea [12], coffee beans [13], Houttuyniae Herba [14], and Ilex Kudingcha C. J. Tseng [15], greatly alleviating the shortage of reference materials and high purchase cost, as well as reducing the workload.

We have reported a QAMS-based HPLC/UV method to analyze nucleosides in Rhizoma Paridis [11]. In this work, a QAMS-based UPLC/MS method was developed for simultaneous determination of nine nucleosides in *T. matsutake*, including adenosine (A), cytidine (C), guanosine (G), inosine (I), uridine (U), 2′-deoxyadenosine (dA), 2′-deoxycytidine (dC), 2′-deoxyguanosine (dG), and 2′-deoxyuridine (dU) (Figure S1). Guanosine was set as the internal reference substance, whose concentration in *T. matsutake* was determined using an external standard. The concentration's relative correction factors (RCFs) between guanosine and eight other nucleosides were determined using external standards. This method was validated to be sensitive, precise, and accurate with the LODs of 0.31–1.9 ng, the overall intraday and interday variations less than 4.08%, and the overall recovery over 89.0%. The correlation coefficients (*r*^2^) of the calibration curves were higher than 0.9918. Then, the concentrations of the components were calculated with the obtained RCFs by QAMS. Finally, vector angle cosine analysis showed that there was no significant difference in concentrations between the external standard method and QAMS, indicating that QAMS can be applied to the multi-indicator quality evaluation of *T. matsutake*.

## 2. Material and Methods

### 2.1. Materials and Instruments

Ten dried samples of *T. matsutake* were collected in accordance with official sampling requirements from Xiaojin County and Jiulong County in Sichuan Province, China, and were labeled SD-JL256, SD-XJ266, SD-JL243, SD-XJ260, SD-JL235, SD-JL244, SD-JL250, SD-XI268, SD-XJ278, and SD-XJ287. Nine authentic standards whose structures are shown in Figure S1 were obtained from Sigma-Aldrich (St. Louis, MO, USA). A Milli-Q water purification system was used to prepare ultrapure water for the UPLC analysis (Millipore, Bedford, MA, USA). Methanol of LC-MS grade and other chemicals/solvents of analytical grade were purchased from Sinopharm Chemical Reagent Co. Ltd. (Shanghai, China). The Bear pulverizer (FSJ-A03D1) purchased from Xiaoxiong Electric Appliance Co., Ltd, Foshan, China, is used for sample crushing. The heated ultrasonic bath (model: AS10200A, volume: 10 L, frequency: 40 kHz, and maximum power: 300 W) purchased from Tianjin Automatic Science Instrument Co., Ltd., (Tianjin, China) was used for sample extraction.

### 2.2. UPLC-MS Analysis

Chromatographic analysis was carried out on a Waters ACQUITY UPLC I-class/XEVO TQ-XS system (Waters, MA, USA). Three analytical columns were used for sample separation: Waters BEH C18 (2.1 mm × 100 mm, 1.7 *μ*m), Agilent ZORBAX SB-C18 (2.1 mm × 100 mm, 1.8 *μ*m), and SHIMADZU Shim-pack XR-ODS (2.0 mm × 100 mm, 1.8 *μ*m). Chromatographic separation was performed on the binary mobile phase system with water (solvent A) and methanol (solvent B) using a gradient of 2%–5% B from 0 to 5 min and 5%–30% B from 5 to 10 min followed by a hold at 30% B, a flow rate of 0.2 mL/min, column temperature of 40°C, and an injection volume of 10 *μ*L.

Target analytes were measured using the multireaction monitoring mode on a triple quadrupole mass spectrometer (triple quad, QqQ MS) equipped with an electrospray ionization source (XEVO TQ-XS; Waters) in negative ion mode. Detector parameters were the source temperature of 150°C, desolvation temperature of 350°C, desolvation gas flow at 800 L/h, cone gas flow at 150 L/h, cone voltage of 40 V, and capillary voltage of −1000 V. The target analytes were measured in the multireaction monitoring mode. The triple quad parameters were automatically optimized using the Tune software (MassLynx V4.2). The ion pairs of the precursor/product ion, cone voltage, and collision energy for each analyte are presented in Table 1.

### 2.3. Sample Preparation

The pretreatment step was carried out according to the routine operation of natural component analysis. The ultrasonic extraction method was applied for sample preparation following the procedures reported previously [16–19]. After crushing and sieving through a No. 4 sieve, 0.5 g of *T. matsutake* was precisely weighed and delivered to a 100 mL volumetric flask. About 80 mL of purified water was added, and the flask was mixed in a heated ultrasonic bath for 30 min. Only the ultrasonic system was used in this experiment. After cooling to room temperature, the flask was filled to the mark with purified water, capped, and inverted to mix. The resultant extract was filtered through a 0.22 *μ*m microporous membrane to prepare the filtrate for the UPLC analysis.

A comparative extraction experiment was performed: six-replicate 0.5 g samples of *T. matsutake* were weighed, three were extracted with 100 mL of water at room temperature by ultrasound, and the other three were extracted with 100 mL of boiling water. The contents of nine components of the extractions were recorded using the same chromatographic conditions.

### 2.4. Preparation of Standard Solution

Individual stock solutions were prepared for the nine nucleosides using authentic standards and purified water as the diluent and stored at 4°C with concentrations of C = 5.75 mg/mL, dC = 4.40 mg/mL, U = 1.20 mg/mL, dU = 4.58 mg/mL, I = 0.928 mg/mL, G = 0.987 mg/mL, dG = 0.877 mg/mL, A = 1.12 mg/mL, and dA = 5.75 mg/mL. An aliquot (1.0 mL) of each stock solution was delivered to a single 100 mL volumetric flask and diluted to volume with purified water to prepare the mixed standard working solution I. An aliquot (1.0 mL) of the mixed standard working solution I was delivered to a single 100 mL volumetric flask and diluted to volume with purified water to prepare the mixed standard working solution II. The mixed standard working solution II was then further diluted 2.5, 5, 10, 20, 50, and 100 times with purified water to prepare the mixed standard working solutions III–VIII, respectively. All standard solutions were stored at 4°C until usage.

### 2.5. Principle and Calculation Method of QAMS

An underlying principle of chromatography is that the response factor (*f*) is a proportional ratio of the detector response, area (A), to the concentration (C) of the analyte (*f* = *A*/*C*) over a certain linear range. Furthermore, in QAMS, one member of a group of related components is selected as the internal standard to establish the RCFs (*f*_*km*_) between that component and other components as follows:

(1) $$\begin{array}{c} f_{km}=\frac{f_{k}}{f_{m}}=\frac{C_{k}\times A_{m}}{C_{m}\times A_{k}}, \end{array}$$

where *f*_*k*_ and *f*_*m*_ are the response factors of the internal standard component and target components, respectively. *C*_*k*_ and *C*_*m*_ are the concentrations of the internal component and other components, respectively, and A_*k*_ and A_*m*_ are the areas of the internal component and other components, respectively. The *f*_*km*_ is then used to calculate the concentrations of the other components as follows:

(2) $$\begin{array}{c} C_{m}=\frac{C_{k}\times A_{m}}{f_{km}\times A_{k}}, \end{array}$$

### 2.6. Similarity Evaluation Based on Vector Angle Cosine Method

The similarity evaluation between QAMS method and external standard method was carried out by vector angle cosine analysis [20]. A cosine ratio value (Cos *θ*) is a vector that calculates the angle between two groups of variables in Euclidian geometry. The closer the Cos *θ* is to 1, the more similar the groups are. Cos *θ* is defined as

(3) $$\begin{array}{c} Cos\;\theta =\frac{\sum_{k=1}^{n}X_{ik}\cdot X_{rk}}{\sqrt{\left(\sum_{k=1}^{n}X_{ik}^{2}\right)\left(\sum_{k=1}^{n}X_{rk}^{2}\right)}}, \end{array}$$

where *X*_*ik*_ is the value of variable *k* in sample *i* and *X*_*rk*_ is the value of variable *k* in common mode.

## 3. Results and Discussion

### 3.1. Optimization of Sample Preparation

We used boiling water to extract nucleosides from *T. matsutake*, as previously described [7, 21]. A room temperature ultrasonic extraction method is more commonly reported for the extraction of nucleosides in the literature [16–19], perhaps due to the convenience of the method. For confidence in the boiling water method, we carried out a comparative experiment by six replicate. The results of the six samples (three from each extraction method) are given in Table 2. Through one-way ANOVA, the results showed no significant difference between the two extraction methods (*P*-value = 0.99). The subsequent method validation results further verified that both methods can be used to extract nucleosides from *T. matsutake.* Therefore, a more convenient ultrasonic extraction method for sample preparation was used in this work.

### 3.2. Establishment of the Improved QAMS Method

**Figure 1:**
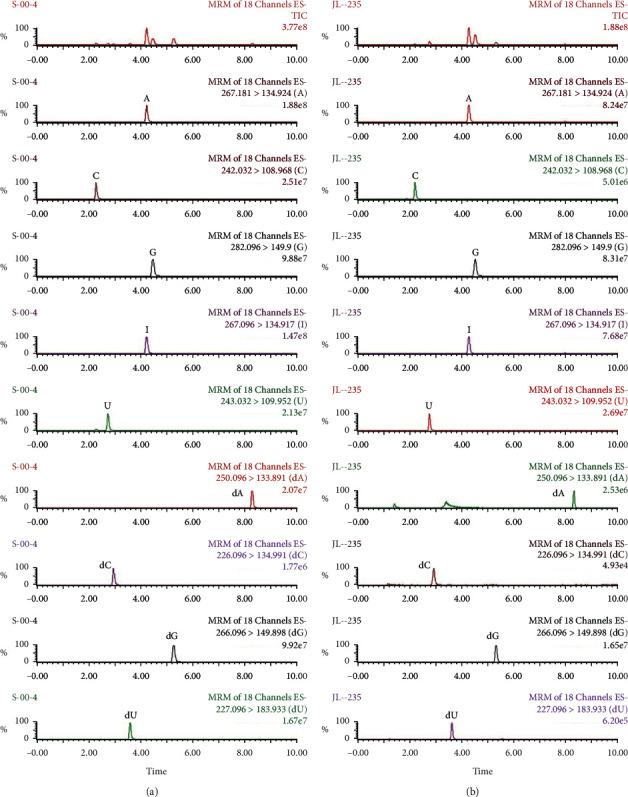
Representative mass spectrum of nine nucleosides in the standard solution (a) and the sample solution (b) using BEH C18 analytical column.

**Figure 2:**
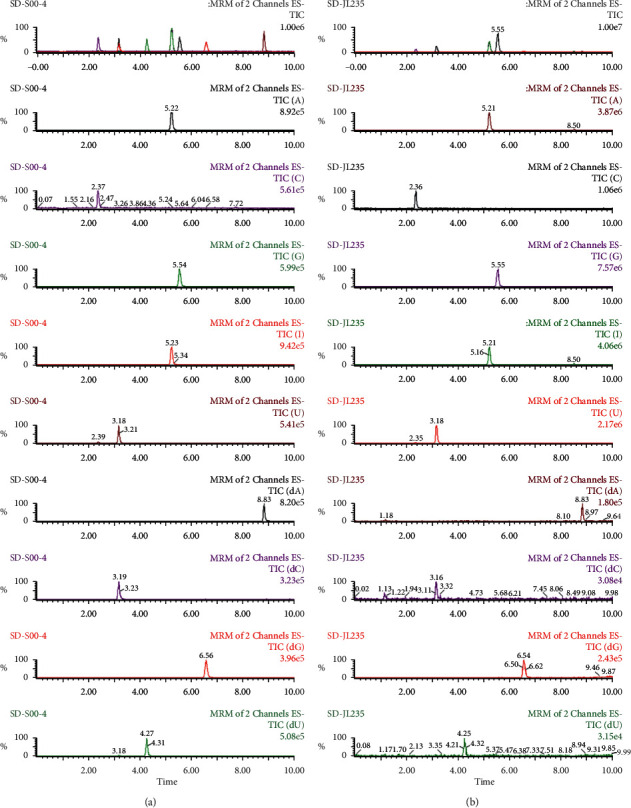
Representative mass spectrum of nine nucleosides in standard solution (a) and sample solution (b) using Agilent ZORBAX SB-C18 analytical column.

**Figure 3:**
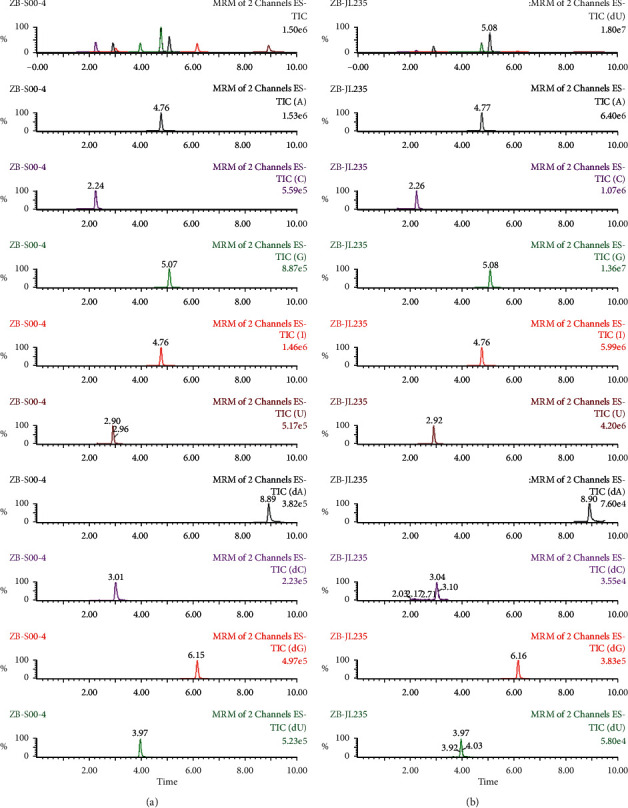
Representative mass spectrum of nine nucleosides in standard solution (a) and sample solution (b) using SHIMADZU Shim-pack XR-ODS analytical column.

After extraction, experiments were performed to optimize the UPLC/MS method. A total of 17 nucleoside compounds have previously been successfully separated using a porous graphitic carbon column by our team [7, 17]. Since the use of porous graphitic carbon columns is not common, conventional C18 analytical columns were selected in this work for the purpose of developing a QAMS UPLC/MS method with good universality. Due to manufacturing differences, including carbon loads, specific surface areas, and end capping methods, even the same types of analytical columns from different manufacturers may have significantly different separation effects, yielding different symmetry factors, retention times, and numbers of theoretical plates. In this work, the chromatographic behaviors of nucleosides were studied on three columns representing a fairly broad range of C18 analytical columns to evaluate the robustness of the QAMS method. The representative mass spectrum of analytes using a Waters BEH C18 analytical column is shown in Figure 1. The chromatograms generated from the other two columns (Agilent ZORBAX SB-C18 and SHIMADZU Shim-pack XR-ODS) are shown in Figures 2 and 3, respectively.

The location of the chromatographic peaks in the chromatogram in terms of the distance between the internal standard peak and the target component peak is a key issue in the success of the QAMS method. The use of the relative retention time (RRT) of the two peaks is adopted in various literature reports, including schemes using the difference and the ratio between the retention times. There are certain shortcomings in the universality of these schemes, despite their simplicity in operation. If the retention time between the internal standard and the target component differs greatly, the relative standard deviation (RSD) of the relative correction factor (RCF) of the difference and the ratio increases. This situation is likely to occur in different instruments, especially in different chromatographic columns, such as in this study. Once beyond the reasonable allowable range, the universality of QAMS method will be reduced. In order to solve this problem, calibration must be carried out using authentic external standards [22], with retention time error < ±1 min and HR-MS data error < ±5 ppm [23, 24].

To investigate the influence of analytical columns on the ruggedness of the RCF values, three columns, i.e., Waters BEH C18, Agilent ZORBAX SB-C18, and SHIMADZU Shim-pack XR-ODS, were tested. The RCF for each of the eight target components relative to guanosine was calculated according to equation (1), with results presented in Table 3. The RSD ranged from 2.67 to 4.87, indicating that RCFs are relatively stable on different analytical columns and can be used for calculation in the QAMS method.

### 3.3. Determination by External Standard Method

The Waters BEH C18 column was used to verify the results using 10 *µ*L injections of the mixed standard working solutions I–VIII as external standards. Regression curves were obtained by binary linear regression analysis for each of the nine authentic standards. The working standard solution was further diluted and injected and the signal-to-noise ratio (S/N) of the peaks was measured to determine the limit of detection (LOD, S/N = 3) and the limit of quantitation (LOQ, S/N = 10) according to the technical guide of Chinese Pharmacopoeia [25] for each of the nine target components. Both intraday precision and interday precision were evaluated as indicators of robustness. Mixed standard working solution III was injected every 4 h for 16 h and again after 24 h, and the RSDs of the average results for the nine components were used to determine the intraday precision. In addition, the standard was injected two times in the morning and again in the evening for three consecutive days, and the RSDs of the average results were used to determine the interday precision of the method for the nine components. Repeatability was tested using six replicate preparations of SD-JL243 and evaluating the RSDs of the average results for the nine components. Recovery was evaluated by spiking the six replicates of SD-JL243 with the standards at an addition ratio of about 1 : 1 to the native concentrations, running the spiked samples on the chromatographic system, calculating the concentrations, and determining the percentage recovery. The method verification data are summarized in Table 4. The results show that the test method meets the standard of quantitative analysis and can be used for the determination of nucleoside compounds in *T. matsutake*. For comparison and confirmation, the contents of the nine nucleoside components in the 10 batches of *T. matsutake* were determined using the external standard method, as given in Table 5.

### 3.4. Determination by QAMS Method

The QAMS method was then used to analyze the 10 batches of *T. matsutake*, using guanosine as the internal compound. The concentrations of the other eight nucleoside compounds in the 10 batches were calculated by equation (2) and are listed in Table 5. Interestingly, the results show that the contents of nucleosides in *T. matsutake* from different regions are quite different, and this may be relevant to their selection for use. From the results of this study and literature reported [7], it is necessary to increase the number of samples in the next study to obtain more data and take the nucleoside as a marker to set up quality control standards for *T. matsutake*.

The similarity evaluation between QAMS method and external standard method was carried out by vector angle cosine analysis, which is a commonly used parameter in the similarity evaluation of Chinese herbal medicine fingerprints [20]. A cosine ratio value (Cos *θ*) is a vector that calculates the angle between two groups of variables in Euclidian geometry. As shown in Table 4, the cosine ratio values were above 0.99845, which indicates no significant differences between the two methods.

## 4. Conclusion

In this study, a QAMS method suitable for the analysis of the nine nucleoside components in *T. matsutake* was established, including adenosine (A), cytidine (C), guanosine (G), inosine (I), uridine (U), 2′-deoxyadenosine (dA), 2′-deoxycytidine (dC), 2′-deoxyguanosine (dG), and 2′-deoxyuridine (dU). Using guanosine as the internal reference, the RCFs for eight other nucleosides were calculated, and then the nucleosides in 10 different samples of *T. matsutake* were quantitatively analyzed. The angle cosine method was used to evaluate the results, and it showed that there was no significant difference in results using the QAMS method or the conventional external standard method. A QAMS method has been established for the determination of the nine nucleoside components in *T. matsutake*, which provides a new choice for the quality evaluation of *T. matsutake*. As far as we know, this is also the first report of the quantitative analysis of nucleoside compounds by UPLC/MS-based QAMS, which can provide an efficient and feasible quality assessment method for other natural products containing nucleosides.

## Figures and Tables

**Table 1:** QqQ/MS parameters for the analysis of nine nucleosides.

Analyte	Parent ion	Product ion	Cone voltage (V)	Collision energy (eV)
Adenosine (A)	267	108^#^	38	38
		135^*∗*^	38	20

Cytidine (C)	242	109^*∗*^	6	12
		152^#^	6	12

Guanosine (G)	282	133^#^	20	32
		150^*∗*^	20	18

Inosine (I)	267	92^#^	40	36
		135^*∗*^	40	22

Uridine (U)	243	110^*∗*^	20	14
		152^#^	20	12

2′-deoxyadenosine (dA)	250	134^*∗*^	34	18
		160^#^	34	22

2′-deoxycytidine (dC)	226	93^*∗*^	6	16
		135^#^	6	16

2′-deoxyguanosine (dG)	266	133^#^	34	18
		150^*∗*^	34	30

2′-deoxyuridine (dU)	227	94^#^	10	18
		184^*∗*^	10	12

^*∗*^quantitative ion, ^#^qualitative ion.

**Table 2:** The results of the comparative study on extraction methods (*μ*g/g).

Extraction methods	A	C	G	I	U	dA	dC	dG	dU
Ultrasonic	44.8 ± 0.5	151.7 ± 1.2	185.9 ± 0.2	37.0 ± 0.8	108.7 ± 0.6	6.6 ± 0.2	13.6 ± 0.4	9.6 ± 0.1	4.1 ± 0.1
Boiling water	45.1 ± 0.4	152.5 ± 1.7	188.1 ± 1.0	36.7 ± 0.2	108.6 ± 0.9	6.5 ± 0.2	13.4 ± 0.2	9.5 ± 0.2	4.2 ± 0.0

**Table 3:** Relative correction factors (RCFs) of nine nucleosides with different analytical columns.

UPLC system	Analytical column	RCFs								
		C	U	dU	I	G	dG	dC	A	dA
Waters UPLC I class	BEH C18	0.129	0.447	0.126	1.925	1	0.636	0.099	1.598	0.107
	SB-C18	0.121	0.438	0.122	2.002	1	0.684	0.102	1.594	0.109
	XR-ODS	0.128	0.480	0.129	1.876	1	0.688	0.107	1.491	0.114
Mean		0.126	0.455	0.125	1.934		0.669	0.103	1.561	0.110
SD		0.004	0.022	0.003	0.063		0.029	0.004	0.061	0.004
RSD		3.45	4.87	2.67	3.28		4.28	3.99	3.90	3.40

**Table 4:** The results of method validation.

Analyte	Regression equation (*y* = *ax* + *b*, *r*^2^)	Linear range (ng/mL)	LOD (ng)	LOQ (ng)	Precision (RSD, *n* = 6)		Repeatability (*n* = 6)		Recovery (*n* = 6)	
					Intraday (%)	Interday (%)	Mean (*μ*g/g)	RSD (%)	Mean (%)	RSD (%)
G	*y* = 6564.29*x* − 354.886, 0.9998	0.9868–98.68	0.3	0.9	2.73	4.08	152.1	0.91	101.9	2.10
A	*y* = 9880.28*x* + 530.894, 0.9991	1.1152–111.52	0.4	1.1	1.26	1.30	108.7	0.61	91.1	2.45
U	*y* = 2565.74*x* + 890.19, 0.9983	1.2–120	0.4	1.2	0.32	0.86	4.2	2.45	88.4	3.78
C	*y* = 772.496 *x* + 1260.27, 0.996	5.742–574.2	1.9	5.7	0.47	1.91	36.9	1.41	97.0	1.76
I	*y* = 11955*x* + 391.889, 0.9989	0.9276–92.76	0.3	0.9	0.55	1.57	187.0	0.74	95.9	2.24
dG	*y* = 4320.97*x* − 506.873, 0.9997	0.8768–87.68	0.3	0.9	0.78	2.14	9.6	1.42	90.9	2.60
dA	*y* = 584.119 *x* + 974.144, 0.9918	5.752–575.2	1.9	5.7	1.41	3.12	13.5	2.38	89.0	2.81
dU	*y* = 839.666 *x* + 943.893, 0.9965	4.58–458	1.5	4.5	2.80	3.22	44.9	0.99	104.7	2.01
dC	*y* = 548.202 *x* + 664.266, 0.9930	4.402–440.2	1.5	4.5	0.58	2.37	6.6	2.47	89.9	2.52

**Table 5:** The contents of nine nucleosides in the 10 batches of *T. matsutake* by the QAMS method and the external standard (ES) method (*μ*g/g).

	C		U		dU		I		G	dG		dC		A		dA	
	ES	QAMS	ES	QAMS	ES	QAMS	ES	QAMS	ES	ES	QAMS	ES	QAMS	ES	QAMS	ES	QAMS
SD-JL256	107.1	106.0	88.9	87.0	4.2	4.1	33.3	31.7	132.4	7.9	7.8	9.3	8.9	40.7	39.1	3.1	3.5
SD-XJ266	129.8	126.0	165.3	160.3	—	—	52.5	49.7	166.4	8.1	8.0	10.4	9.7	63.6	62.3	4.2	4.2
SD-JL243	152.9	152.0	108.6	108.0	4.0	3.9	36.4	34.6	186.1	9.6	9.6	13.1	12.8	45.2	44.2	6.8	6.8
SD-XJ260	123.2	122.5	126.7	123.7	—	—	34.2	32.6	131.2	7.4	7.3	10.1	9.6	41.6	39.0	3.9	3.5
SD-JL235	96.3	92.0	91.5	79.8	—	—	36.8	35.0	127.5	5.9	5.8	7.4	6.9	44.4	42.6	1.3	1.3
SD-JL244	85.2	91.9	93.3	90.8	4.0	3.9	34.9	33.2	104.9	4.2	4.0	3.4	3.1	41.9	39.3	—	—
SD-JL250	92.7	88.8	101.4	98.4	—	—	39.8	37.8	129.1	5.7	5.6	5.6	5.4	48.2	46.1	—	—
SD-XJ268	76.0	74.2	122.3	120.6	—	—	37.9	36.0	95.7	4.0	3.8	1.3	1.4	45.6	44.7	—	—
SD-XJ278	102.0	99.3	191.3	186.1	—	—	67.7	64.0	141.2	5.5	5.4	5.2	5.0	81.5	80.7	—	—
SD-XJ287	75.3	73.6	123.9	121.3	4.1	4.1	40.5	38.5	90.1	4.0	3.8	1.3	1.4	50.0	48.7	—	—

Cos *θ*	0.99960		0.99969		0.99996		1.00000			0.99989		0.99974		0.99985		0.99845	

## Data Availability

The data used to support the findings of this study are available from the corresponding author upon request.

## Supplementary Materials

Supplementary Materials Figure S1. Chemical structures of nine nucleosides determined in *T. matsutake*.
