# Supplementary material for: A Practical Method for Determination of Nine Nucleosides in Tricholoma matsutake by UPLC/MS and Quantitative Analysis of Multicomponents Using Single Marker Method
Source: J Anal Methods Chem. 2021 Sep 11;2021:9571329. doi: 10.1155/2021/9571329 (PMC8452435; doi:10.1155/2021/9571329)
Supplement: Supplementary Materials — Figure S1. Chemical structures of nine nucleosides determined in T. matsutake. [file 9571329.f1.docx]

**Supplementary Material of the Manuscript**

**A practical method for determination of nine nucleosides in *Tricholoma matsutake* by UPLC/MS and quantitative analysis of multicomponents using a single marker method**

Li Yong, An-Qin Leng, Zhi-Xiang Yang, Ying Xue

*Sichuan Provincial Center for Disease Control and Prevention, Chengdu 610041, China*

Correspondence should be addressed to Ying Xue, xuecher0221@sina.com





Figure S1. Chemical structures of nine nucleosides determined in *T. matsutake*
